# Wave breaking field of relativistically intense electrostatic waves in electronegative plasma with super-thermal electrons

**DOI:** 10.1038/s41598-022-16481-z

**Published:** 2022-07-18

**Authors:** Arghya Mukherjee

**Affiliations:** ELI Beamlines Centre, Institute of Physics, Czech Academy of Sciences, Za Radnicí 835, 25241 Dolní Břežany, Czech Republic

**Keywords:** Mathematics and computing, Physics

## Abstract

The wave breaking limit of relativistically intense electrostatic waves in an unmagnetised electronegative plasma, where electrons are alleged to attach onto neutral atoms or molecules and thus forming a significant amount of negative ions, has been studied analytically. A nonlinear theory has been developed, using one-dimensional (1D) relativistic multi-fluid model in order to study the roles of super-thermal electrons, negative ion species and the Lorentz factor, on the dynamics of the wave. A generalised kappa-type distribution function has been chosen for the velocities of the electrons, to couple the densities of the fluids. By assuming the travelling wave solution, the equation of motion for the evolution of the wave in a stationary wave frame has been derived and numerical solutions have been presented. Studies have been further extended, using standard Sagdeev pseudopotential method, to discover the maximum electric field amplitude sustained by these waves. The dependence of wave breaking limit on the different input parameters such as the Lorentz factor, electron temperature, spectral index of the electron velocity distribution and on the fraction and the mass ratio of the negative to positive ion species has been shown explicitly. The wavelength of these waves has been calculated for a wide range of input parameters and its dependence on aforementioned plasma parameters have been studied in detail. These results are relevant to understand particle acceleration and relativistic wave breaking phenomena in high intensity laser plasma experiments and space environments where the secondary ion species and super-thermal electrons exist.

## Introduction

The interaction of ultrashot, ultraintense electromagnetic pulse with a plasma often offers a bunch of interesting nonlinear physical phenomena^1–4^. Concurrently, the amelioration in the high intensity laser science and technology in the last few decades, specifically in producing shorter pulses^5–8^, has made it possible to discover a new regime of physics with many applications^3,9^. Depending on the pulse and the plasma parameters, the interaction of ultrashort, high-intensity laser pulses may trigger a wide variety of nonlinear physical effects. Generation of nonlinear plasma waves in the wake of the laser is one of these collective plasma excitations. The nonlinear plasma waves generated either by a high intensity laser pulse^10,11^ (or, a ultrarelativistic beam pulse^12,13^) in a plasma are capable of supporting very high electric field amplitude over a very small spatial scale. Charged particles injected properly into the wake may get trapped in the wave and can be accelerated to very high energies. From recent experiments^14–19^ it has been found that laser-plasma accelerators can provide an energy gain of almost 1000 times higher than the maximum energy gain threshold of a conventional RF accelerator, due to the their thermionic breakdown. These higher energetic particles are crucially desired in laser assisted fast ignition fusion devices^20^ and in bio-medical industries^21^. As a result, the study of laser/beam driven nonlinear plasma waves and associated particle acceleration mechanisms have been receiving constantly growing interest in recent years.

But in reality, these wake waves can not accelerate the particles to infinite energies^22^. The maximum acceleration rate is actually controlled by the maximum electric field amplitude sustained by the wave over a sufficient time, which is typically known as the “wave breaking limit”^23^. This wave breaking in a plasma may occur through a variety of nonlinear effects. In 1959, Dawson^23^ first pioneered the concept of wave breaking for nonlinear, nonrelativistic electron plasma oscillations in a cold plasma. By considering the electrons as an infinite sheet of moving charges along one dimension and treating the ions as stationary neutralizing background, Dawson derived that the maximum electric field amplitude generated via nonlinear, nonrelativistic plasma oscillations in a cold plasma is limited by $E_{D} = m_e\omega _{pe}v_{ph}/e$, where *e* and $m_e$ respectively be the charge and mass of the electrons, $v_{ph}$ is the phase velocity and $\omega _{pe}$ is the electron plasma frequency, given by $\omega _{pe} = \sqrt{4\pi n_0e^2/m_e}$. Physically it is not possible to excite plasma oscillation beyond this critical limit as the crest of the wave appears to overturn. Physically the trajectory of the neighbouring electrons constituting the oscillation/wave start to cross each other within a plasma period which ultimately destroy the coherence of the wave, manifested by multi stream flow and density singularity^23^.

Now, in typical laser/beam-plasma accelerator experiments^13–17,22^, the amplitudes of these nonlinear plasma waves reach so high that the work done by the electric field of the wave within one wavelength becomes comparable to the rest mass energy of the oscillating particles. In these situations oscillations/waves become relativistically intense and relativistic mass variation effect of the particles becomes influencial^22^. The maximum amplitude sustained by relativistically intense plasma waves, supported by electron motion, was calculated earlier than Dawson’s^23^ discovery of wave breaking for nonrelativistic plasma oscillations. In 1956, Akhiezer and Polovin^24^ obtained an exact one-dimensional longitudinal travelling wave solution in a cold plasma, including relativistic mass effects of the electrons. This travelling wave solution is obtained by solving the cold plasma relativistic fluid-Maxwell set of equations in a stationary wave frame. It has been derived that maximum electric field amplitude $(E_{AP})$ of this wave is limited by the following expression $E_{AP} = \frac{m_e\omega _{pe}c}{e}\sqrt{2(\gamma _{ph} - 1)}$, where *c* is the velocity of light in free space and $\gamma _{ph} = 1/\sqrt{1 - (v_{ph} ^2/c^2)}$ the Lorenz factor. The effect of finite electron temperature on the wave breaking limit for nonrelativistic electron plasma wave was studied by Coffey^25^ in 1971 by using a “water-bag” distribution for electrons and this calculation has been extended later by several authors in the relativistic regime^26–29^. In both nonrelativistic^25^ and relativistic regime^26–29^, the inclusion of finite electron temperature results in decreasing the wave breaking limit in comparison with their respective cold plasma wave breaking amplitude. In all these aforementioned references^24–30^, the motions of the ions are not taken into account.

The influence of ion motion on the dynamics of relativistically intense electrostatic plasma waves was first presented by Khachatryan^31^ in 1998. From the relativistic two fluid model it has been shown that although the wave-breaking field weakly depends on the mass of ions, but the wavelength of the nonlinear waves essentially changes significantly as a result of ion’s response^31^. Therefore, the maximum energy of the accelerated particles also gets modified. Physically in a electron-ion plasma as the mass ratio of electron to ion decreases, the wave breaking field also decreases^31,32^. It has also been found that, the presence of a secondary ion species in an electron-ion plasma explicitly changes the dynamics of the wave and also modifies the wave breaking field^33^. Recently, the self-injection of ions and generation of higher energetic ions pulses have been studied via ion wave breaking mechanism for laser intensities in the range of $10^{20}{-}10^{23} \;\text{W}/\text{cm}^2$^34^. This mechanism is similar to the ion acceleration in the bubble regime, where the bubble changes to a double layer wake configuration^35–37^. It has been found that since the self-injection of ions leads to the annihilation in downstream part of the bubble^34^, a multi-ion species plasma can be used in order to increase the acceleration of the ions^35,37^. In this multi-ion species plasma, all lighter ions are trapped and heavy ions are responsible for the stabilizing background. From extensive numerical simulations, it has also been observed that the acceleration is enhanced when there a presence of heavier ions of sufficient fraction^37^.

Very recently (in 2021), an analytical expression for the wave breaking amplitude of nonlinear electrostatic waves, in a electronegative plasma (where heavier negative ions are present), in the presence of Kappa ($\kappa$)-distributed^38^ electrons has been reported by Elkamash et al.^39^. It has been theoretically found that the presence of negative ion-species enhances the wave breaking amplitude [Figure 2.(b) and 3.(b) in Ref.^39^]. Naturally, these results are highly relevant to particle acceleration in plasma based accelerators and also in the space environment, where multi-ion species plasmas are regularly encountered^40,41^. But, for present day plasma accelerators where high intensity lasers are being used, relativistic effects may govern the wave dynamics which may lead to a modification in the wave breaking amplitude of electrostatic waves in electronegative plasmas.

Here we note that there exists a number of systems where the ions are treated relativistically under certain conditions^34–37,42–44^. For example, in a typical two component hydrogen plasma, ions start behaving relativistically when $\gamma _{ph} > \left( \frac{M_i}{16m_e}\right) ^{1/3}$; where $M_i$ and $m_e$ respectively be the ion and electron mass^31^. In the other hand, propagation of intense short laser pulses in a plasma (or, interaction of intense laser pulse with a thin target having high atomic number) can also lead to pair production which eventually results in formation a multi component plasma, where ions can be treated realtivistically^40,42,43,45^.

Driven by the above motivations, in this manuscript, we present a theory of the electrostatic wave dynamics in an unmagnetised electronegative plasma, by treating the ion species relativistically, and discover its wave breaking amplitude. Such negative-ion plasmas are usually observed in various environments in space and in the laboratory^46–53^. At the outset in “The relativistic multi fluid model and linear dispersion relation” section, we furnish the normalised set of equations for three different plasma components—positive ions, negative ions and electrons. Electronagetive plasmas are often characterized by the presence of superthermal electrons^38,41^, thus here we have used a Kappa $(\kappa )$-type distribution for electrons. We also present the linear dispersion relation of these waves in this section. Next, in “Nonlinear analysis: travelling wave approximation and solutions in the stationary frame” section, we provide the equation of the dynamics of the wave in a stationary frame of reference and present its numerical solutions. Then in “Determination of the wave breaking field: pseudopotential method” section, we derive the wave breaking limit using standard Sagdeev pseudopotential approach. We also study the dependence of the wavelength on different input plasma parameters in this section. Finally, we consolidate our work and conclude in “Conclusions” section.

## The relativistic multi fluid model and linear dispersion relation

Here we consider a collisionless, unmagnetised, homogeneous plasma comprising two different cold ion species, with positive and negative charges, and negatively charged electrons. The population of electrons essentially follows the Kappa distribution. The respective mass and charges of the positive and negative ion species are $m_1$ and $q_1 = z_1e$ and $m_2$ and $q_2 = -z_2e$; *e* denotes the absolute elementary charge, stated earlier. We employ relativistic cold fluid equations for the ions assuming that the ion energies in the wave motion are much larger than the respective ion temperatures. We further assume that the spatial variations of the plasma parameters are along the longitudinal direction only (here along *x* direction). Thus the equation for continuity and the momentum equation for the ion species can be written as

1 $$\begin{aligned}&\frac{\partial n_1}{\partial t} + \frac{\partial }{\partial x}(n_1v_1) = 0 \end{aligned}$$

2 $$\begin{aligned}&\frac{\partial p_1}{\partial t} + v_1\frac{\partial p_1}{\partial x} = -z_1e\frac{\partial \phi }{\partial x} \end{aligned}$$

3 $$\begin{aligned}&\frac{\partial n_2}{\partial t} + \frac{\partial }{\partial x}(n_2v_2) = 0 \end{aligned}$$

4 $$\begin{aligned}&\frac{\partial p_2}{\partial t} + v_2\frac{\partial p_2}{\partial x} = z_2e\frac{\partial \phi }{\partial x} \end{aligned}$$

where, $n_j$ and $v_j$ $(j = 1, 2)$ respectively be the density and velocity of the ion species; $j=1$ for the positive ion species and $j=2$ for the negative ion species. $\phi$ represents the electrostatic potential of the wave and $p_j$ stands for the ion momentum which can be expressed as $p_j = m_j\gamma _jv_j$, where $\gamma _j = \sqrt{1 - \frac{v_j^2}{c^2}}$. In the above fluid equations the densities are in the laboratory frame of reference. These densities $n_1$ and $n_2$ are connected to each other via the Poisson’s equation, which for the above electronegative plasma system stands as

5 $$\begin{aligned} \frac{\partial ^2\phi }{\partial x^2} = -\frac{e}{\epsilon _0}\left( z_1 n_1 - z_2 n_2 - n_e \right) \end{aligned}$$

In the above equation $n_e$ is the density of the electrons and now we need an expression for the number density of the electrons as a function of the potential $\phi$, which is usually evaluated by considering a distribution for the velocities of the electron species. A standard approach^39^ is to take this situation into account by adopting a so-called $\kappa$ distribution function, having the following form^38^

6 $$\begin{aligned} f_e(v_e) = n_{e,0}(\pi \kappa \theta ^2)^{-3/2}\frac{\Gamma (\kappa + 1)}{\Gamma (\kappa - 1/2)}\left( 1 + \frac{v_e^2}{\kappa \theta ^2}\right) ^{-(\kappa + 1)} \end{aligned}$$

where, $$n_{e,0}$$ is the equilibrium electron density and $$\kappa $$ represents the spectral index of distribution and in the limit $$\kappa \rightarrow \infty $$ the above distribution becomes a standard Maxwellian distribution. $$\theta $$ represents the most probable speed which is essentially related to the thermal speed ($$v_{th,e} = \sqrt{2k_BT_{e}/m_e}$$) via the following relation: $$\theta = v_{th,e}\left( \frac{\kappa - 3/2}{\kappa }\right) ^{1/2}$$. The Gamma-functions ($$\Gamma $$) usually come from the normalisation which is given by $$\int _0 ^\infty f_{e}(v_e) d^3v_e = n_{e}$$^54^. In the presence of the wave i.e. for nonzero value of the electrostatic potential, one can integrate Eq. (6) to obtain the number density of the electrons ($$n_e)$$ as

7 $$\begin{aligned} n_e (\phi ) = n_{e,0}\left[ 1 - \frac{e\phi }{k_BT_{e}(\kappa - 3/2)}\right] ^{-\kappa + 1/2} \end{aligned}$$

Now, for the sake of our analytical calculations here we adopt the following normalisation: $t \rightarrow \omega _{p1}t$, $x \rightarrow \frac{x\omega _{p1}}{c}$, $V_j \rightarrow \frac{v_j}{c}$, $p_j \rightarrow \frac{p_j}{m_1c}$, $N_j \rightarrow \frac{n_j}{n_{j,0}}$, $E \rightarrow \frac{z_1eE}{m_1\omega _{p1}c}$, $\phi \rightarrow \frac{z_1e\phi }{m_1c^2}$; $\omega _{p1} = \left( \frac{z_1^2e^2n_{1,0}}{\epsilon _0 m_1} \right) ^{-1/2}$ is the positive ion plasma frequency and $n_{1,0}$ represents the equilibrium value of the positive ions in the absence of the wave. We also introduce five dimensionless parameters in order to understand the parametric dependence of the wave characteristics on the initial plasma parameters. These are as follow: the negative-to-positive ion mass ratio $\left( \mu = \frac{m_2}{m_1} \right)$, the negative-to-positive ion charge ratio $\left( Q = \frac{q_2}{q_1} \right)$, the equilibrium negative-to-positive ion density ratio $\left( \delta _i = \frac{z_2n_{2,0}}{z_1n_{1,0}} \right)$, the equilibrium electron-to-positive ion density ratio $\left( \delta _e = \frac{n_{e0}}{z_1n_{1,0}} \right)$ and normalised electron thermal energy $\left( \beta _{th,e} = \frac{k_BT_e}{m_1c^2} \right)$. We also note that the charge neutrality condition at equilibrium yields

8 $$\begin{aligned} \delta _e + \delta _i = 1 \end{aligned}$$

Using the above normalisation, Eqs. (1–5) and (7) respectively can be transformed to

9 $$\begin{aligned} \frac{\partial N_1}{\partial t} + \frac{\partial }{\partial x}(N_1V_1) = 0 \end{aligned}$$

10 $$\begin{aligned} \left[ \frac{\partial }{\partial t} + V_1\frac{\partial }{\partial x} \right] (\gamma _1V_1) = -\frac{\partial \phi }{\partial x} \end{aligned}$$

11 $$\begin{aligned} \frac{\partial N_2}{\partial t} + \frac{\partial }{\partial x}(N_2V_2) = 0 \end{aligned}$$

12 $$\begin{aligned} \left[ \frac{\partial }{\partial t} + V_2\frac{\partial }{\partial x} \right] (\gamma _2V_2) = \frac{Q}{\mu }\frac{\partial \phi }{\partial x} \end{aligned}$$

13 $$\begin{aligned} \frac{\partial ^2\phi }{\partial x^2} = \delta _eN_e + \delta _iN_2 - N_1 \end{aligned}$$

and

14 $$\begin{aligned} N_e (\phi ) = \left[ 1 - \frac{\phi }{\beta _{th,e}(\kappa - 3/2)}\right] ^{-\kappa + 1/2} \end{aligned}$$

As a first step, we can linearise Eqs. (9–14), to derive the dispersion relation of small amplitude propagating waves having frequency $\omega$ and wavenumber $k_p$, which can be expressed as

15 $$\begin{aligned} \omega ^2 = \frac{k_p^2\left( 1+\delta _i\frac{Q}{\mu }\right) }{k_p^2 + \frac{\delta _e}{\beta _{th,e}}\frac{(\kappa - 1/2)}{(\kappa - 3/2)}} \end{aligned}$$

**Figure 1:**
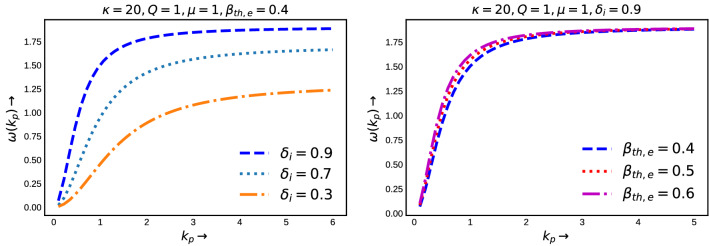
Dispersion curves of electrostatic waves in an electronegative plasma. Left: For different values of negative ion concentration and Right: For different values of electron thermal energy. Other parameters are kept constant as mentioned.

**Figure 2:**
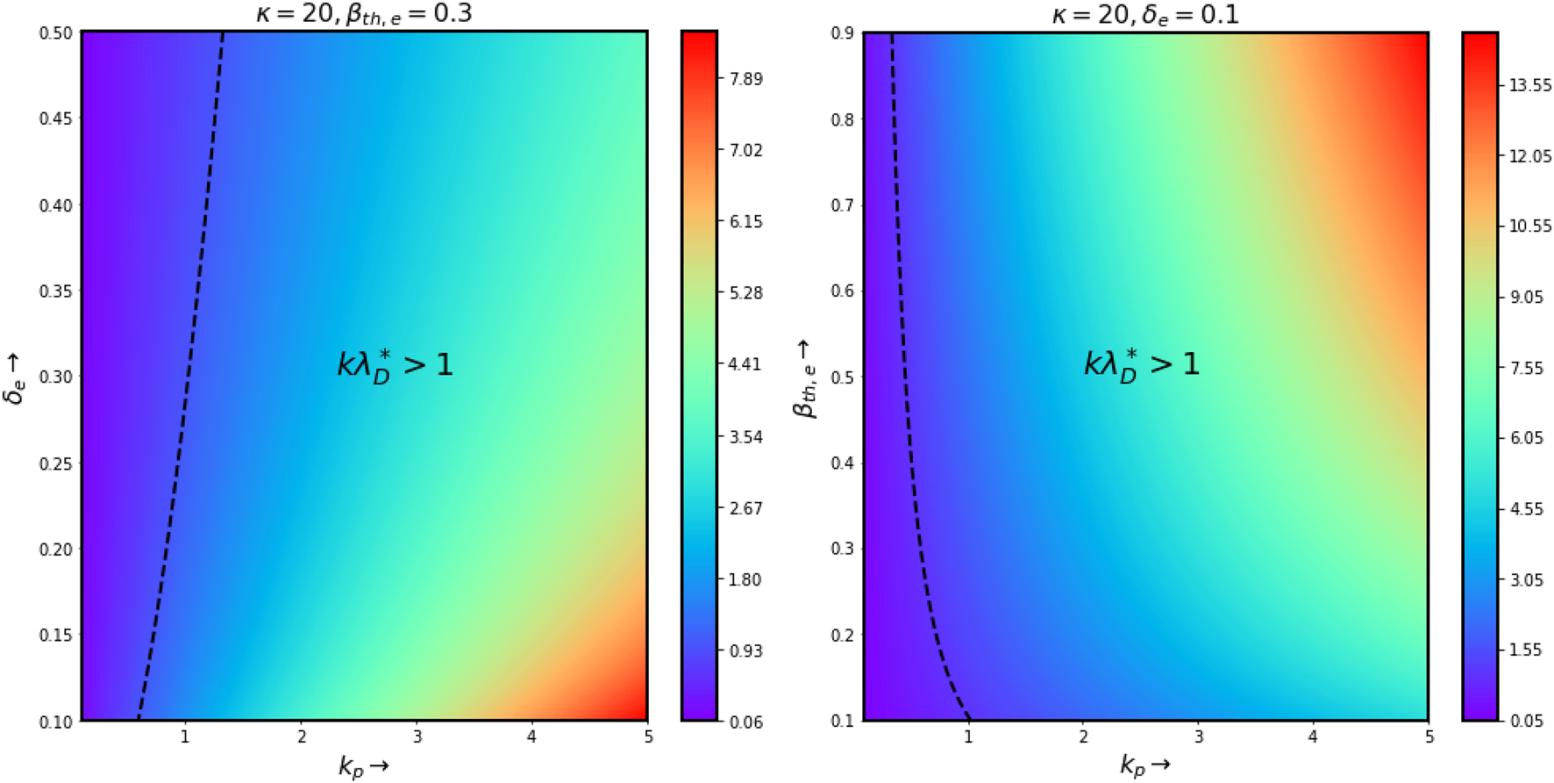
variation of $k_p\lambda _D^\star$ for electrostatic waves in an electronegative plasma. Left: For different values of electron concentration and Right: For different values of electron thermal energy. Other parameters are kept constant as mentioned. The black dotted line represents $k_p\lambda _D^\star = 1$.

The variation of $\omega$ as a function of $k_p$, given by Eq. (15), has been depicted in Fig. 1. From the both two figures, we see that the the frequency increases rapidly for lower values of $k_p$ and then it reaches to an asymptotic value for higher wave number. From these two figures we also see that, in the linear limit, the frequency increases with the concentration of negative ion parameters (left panel of Fig. 1) and electron thermal velocity (for small wave number values in the right panel of Fig. 1), respectively, for fixed values of other input plasma parameters. Now, the above equation can also be expressed as

16 $$\begin{aligned} \omega ^2 = \frac{k_p^2\lambda _D^{\star ^2}\left( 1+\delta _i\frac{Q}{\mu }\right) }{1 + k_p^2\lambda _D^{\star ^2}} \end{aligned}$$

where, $\lambda _D^{\star } = \left[ \frac{\beta _{th,e}}{\delta _e}\frac{(\kappa - 3/2)}{(\kappa - 1/2)} \right] ^{1/2}$ is s the kappa dependent Debye screening length or modified Debye length in a electronegative plasma. Eq. (16) gives the information of the wave dynamics for two limiting cases. Firstly, for $k_p^2\lambda _D^{\star ^2}>> 1$ (when the wavelengths are less than the kappa dependent Debye screening length), $\omega ^2$ converges to the linear ion plasma oscillations. Secondly, for $k_p^2\lambda _D^{\star ^2}<< 1$ (when the wavelengths are greater than the kappa dependent Debye screening length), the dispersion relation represents dispersive ion acoustic mode^55^. Now, in an electronegative plasma the predefined kappa dependent Debye screening length explicitly depends on electron thermal energy and electron populations. In order to understand the effects of electron thermal energy and electron populations on the dispersion curves, here in Fig. 2 we have shown the contour plots of $k_p\lambda _D^\star$. The color represents the value of $k_p\lambda _D^\star$. The black dotted line is representing $k_p\lambda _D^\star = 1$. The region where $k_p\lambda _D^\star > 1$ is also marked (the right hand portion of the dotted black line). From the left panel, we see that for a fixed value of electron temperature, as the electron population increases, $k_p\lambda _D^\star = 1$ straight line also moves towards the higher $k_p$ values. At a specific electron concentration, for all the wave number values, which are on the left hand side of the $k_p\lambda _D^\star = 1$ line, will give rise to ion acoustic mode, whereas the higher wave numbers on the right hand side of the $k_p\lambda _D^\star = 1$ line will lead to the generation of ion oscillations. On the other hand, for fixed values of electron concentration, as the electron temperature increases the $k_p\lambda _D^\star = 1$ curve shifts towards the lower wave number values. It indicates that in an electronegative plasma the ion acoustic modes are more likely to be found for relatively smaller values of $\beta _{th,e}$, so that $k_p\lambda _D^\star$ remains $<< 1$. Later, we will study the dispersion curves for nonlinear amplitudes by measuring the wavelengths of the waves from the solution of equation of motion of the waves.

In the next section we derive the equation of motion by assuming travelling wave approximation and present its numerical solutions in the nonlinear regime.

## Nonlinear analysis: travelling wave approximation and solutions in the stationary frame

In this section we consider travelling wave solutions in a reference frame moving with the phase velocity of the wave ($V_{ph}$, normalised with *c*). Thus we will express all the fluid variables as functions of a single moving coordinate $\psi = x - V_{ph} t$. This assumption allows to replace $\frac{\partial }{\partial t}$ and $\frac{\partial }{\partial x}$ by $-V_{ph}\frac{d}{d\psi }$ and $\frac{d}{d\psi }$, respectively. So, from the continuity equations for the ions, one can write

17 $$\begin{aligned} N_{j = 1,2} = \frac{V_{ph}}{V_{ph} - V_j} \end{aligned}$$

Now Eq. (10) can be written as

$$\begin{aligned} \frac{d}{d\psi } \left[ \frac{1 - V_{ph} V_1}{(1 - V_1^2)^{1/2}} \right] = \frac{d\Phi _1}{d\psi } \end{aligned}$$

where, $\Phi _1 = (1 - \phi ) \ge \frac{1}{\gamma _{ph}}$. By choosing the condition that in the absence of the wave, when $\Phi _1 = 1$ (i.e for $\phi = 0$), $V_1 = 0$, the above equation can be integrated to obtain the expression for $V_1$ as a function of $\Phi _1$ (or $\Phi$), which can be expressed as

18 $$\begin{aligned} V_1 (\phi ) = \frac{V_{ph} - \Phi _1 (\Phi _1^2 - \gamma _{ph} ^{-2})^{1/2}}{(V_{ph} ^2 + \Phi _1^2)} \end{aligned}$$

Similarly, Eq. (12) yields

19 $$\begin{aligned} V_2(\phi ) = \frac{V_{ph} - \Phi _2 (\Phi _2^2 - \gamma _{ph} ^{-2})^{1/2}}{(V_{ph} ^2 + \Phi _2^2)} \end{aligned}$$

where, $\Phi _2 = 1 + \frac{Q}{\mu }\phi = 1 + \frac{Q}{\mu }(1 - \Phi _1) \ge \frac{1}{\gamma _{ph}}$. Here $\Phi _1$ and $\Phi _2$ are defined in terms of $\phi$ just for the sake of calculation and presentation^31^. Now substituting the expressions for $V_{j = 1,2}$, from Eqs. (18) and (19) respectively, into the Eq. (17) we get the expression for $N_{j = 1,2}$ as a function of $\phi$, which take the following form

20 $$\begin{aligned} N_{j = 1,2}(\phi ) = V_{ph} \gamma _{ph} ^2 \left[ \frac{\Phi _{j=1,2}}{(\Phi _{j=1,2}^2 - \gamma _{ph} ^{-2})^{1/2}} - V_{ph} \right] \end{aligned}$$

Now, finally substituting the expressions for $N_{j = 1,2} (\phi )$ and $N_e (\phi )$, from Eqs. (14) and (20) respectively, into Eq. (13), we obtain

21 $$\begin{aligned} \frac{d^2\phi }{d\psi ^2} + V_{ph} \gamma _{ph} ^2 \left[ \frac{\Phi _1}{(\Phi _1^2 - \gamma _{ph} ^{-2})^{1/2}} - V_{ph} \right] - \delta _i V_{ph} \gamma _{ph} ^2 \left[ \frac{\Phi _2}{(\Phi _2^2 - \gamma _{ph} ^{-2})^{1/2}} - V_{ph} \right] - \delta _e\left[ 1 - \frac{\phi }{\beta _{th,e}(\kappa - 3/2)}\right] ^{(-\kappa + 1/2)} = 0 \end{aligned}$$

This equation represents the evolution of the electrostatic wave in an electronegative plasma, in the stationary frame of reference. Here we would like to mention that an exact analytical solution of this equation is not possible as it contains highly nonlinear terms which can not be integrated analytically. Still the nature of the wave profiles can be studied by solving it numerically, using *4-th* order Runge-Kutta scheme, with proper set of initial conditions. Thus, here we present the numerical solution of Eq. (21) and discuss the properties of the waves below.

**Figure 3:**
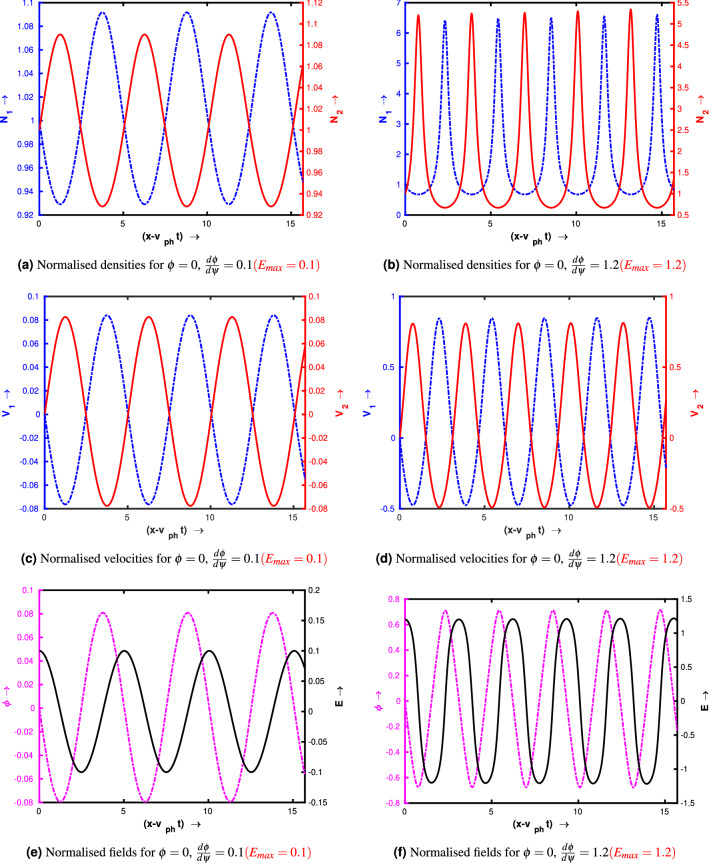
Profile of the wave for $\delta _i = 0.9$, $\gamma _{ph} = 100$, $\beta _{th,e} = 0.3$, $Q = 1$, $\mu = 1$ and $\kappa = 20$.

**Figure 4:**
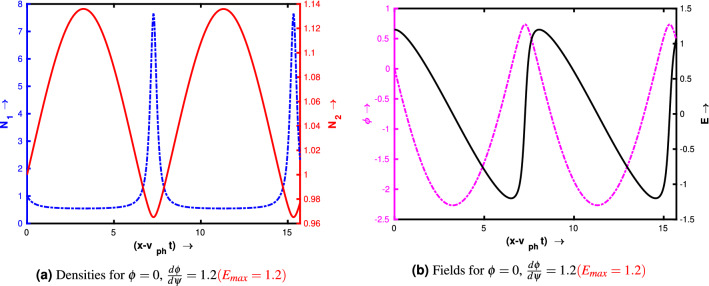
Profile of the wave for $\delta _i = 0.9$, $\gamma _{ph} = 100$, $\beta _{th,e} = 0.3$, $Q = 1$, $\mu = 20$ and $\kappa = 20$.

Figure 3a–f show the profiles of the wave for two different set of initial conditions. For the left panel the initial conditions are $\phi = 0$ and $\frac{d\phi }{d\psi } = 0.1$ at $\psi = 0$ (linear amplitude). For the right panel the initial conditions are $\phi = 0$ and $\frac{d\phi }{d\psi } = 1.2$ at $\psi = 0$ (nonlinear amplitude). The following quantities have been kept constant: $\delta _i = 0.9$, $\gamma _{ph} = 100$, $\beta _{th,e} = 0.3$, $Q = 1$, $\mu = 1$ and $\kappa = 20$. By comparing the figures from the right panel and the corresponding left panel, we see that as the value of $\frac{d\phi }{d\psi }$ increases, the wave electric field steepens due to nonlinearity which is manifested by the nonlinear density spikes. Similar trend can also be seen in the velocity profile also, as the fluid velocity amplitude starts to reach the phase velocity of the wave. These linear and nonlinear amplitudes can be defined by the amplitude of $\frac{d\phi }{d\psi }$. When $\frac{d\phi }{d\psi }<< 1$, the wave is a linear sinusoidal wave. But as $\frac{d\phi }{d\psi }$ increases the crest of the wave becomes spiky, which can be treated as nonlinear waves. In both the panel, the numerical solution has been presented for $\psi = 5\pi$ and we see that the number of the wave crest significantly increases as the value of $\frac{d\phi }{d\psi }$ has been changed from 0.1 to 1.2. As the distance between two consecutive crests represents the wavelength of the wave, thus we may conclude that the wavelength is amplitude dependent which is a characteristic of nonlinear oscillations/waves. The profile of the waves also depends on the ratio of the negative to positive ion mass $\mu$. In Fig. 4 we have shown the numerical solution of Eq. (21) for a higher value of $\mu = 20$. Fig. 4a represents the normalised density profiles and Fig. 4b depicts the normalised electric field and potential profiles. Other input parameters have been kept as same as in Fig. 3. By comparing from the right panel of Fig. 3, we see that for the same values of the initial conditions ($\phi = 0$ and $\frac{d\phi }{d\psi } = 1.2$ at $\psi = 0$), the profile of the waves changes significantly. Even for the same electric field value ($\frac{d\phi }{d\psi } = 1.2$), the amplitude of negative ion density is now very less as compared to Fig. 3b. It happens due to the higher mass of the negative ion species. For linear amplitude limit ($\phi = 0$ and $\frac{d\phi }{d\psi } = 0.1$ at $\psi = 0$), this density amplitude is even more smaller ($10^{-3}$) and thus in this case the higher negative ions are almost stabilized. And for higher amplitude case, the lighter ions are expected to be trapped in the potential on the onset of wave breaking^32,35,37^. We also see that the distance between two consecutive peaks increases as the value of $\mu$ increases from 1 to 20 (this will be discussed in detail in the next section).

Here we would like to mention that these numerical solutions have been obtained by assigning an initial value of $\phi$ ($\phi _0$, say) and corresponding $\frac{d\phi }{d\psi }$ at $\psi = 0$. These two values have been given as an input initial condition, and for a particular numerical run, the values of other plasma parameters like $\delta _i$, $\beta _{th,e}$, $\gamma _{ph}$, *Q*, $\mu$ and $\kappa$ have been kept constant. The values of $\phi$ and $\frac{d\phi }{d\psi }$ at $\psi = 0$ are not arbitrary. For a particular initial value of $\phi$ the corresponding $\frac{d\phi }{d\psi }$ should be taken. This $\frac{d\phi }{d\psi }$ can be obtained by multiplying Eq. (21) by $\frac{d\phi }{d\psi }$ and then integrating over $\psi$. Periodic solutions will be observed provided the amplitude of $\frac{d\phi }{d\psi }$ ($E_{max}$) should be less than the corresponding wave breaking limit. In Figs. 3 and 4 the profiles of the waves have been shown for two different sets of initial conditions $\phi = 0$, $\frac{d\phi }{d\psi } = 0.1$ (i.e.$E_{max} = 0.1$) and $\phi = 0$, $\frac{d\phi }{d\psi } = 1.2$ (i.e. $E_{max} = 1.2$). In both two cases maximum electric field amplitude $E_{max}$ is less than the corresponding wave breaking limit. We note that the initial value $\phi _0$ and the corresponding $\frac{d\phi }{d\psi }$ decide the initial phase of the wave. The maximum amplitude does not depend on the choice of the initial phase of the wave. It means if we use different initial $\phi$ and corresponding $\frac{d\phi }{d\psi }$ (keeping $E_{max}$ as fixed) as initial conditions then the value of the maximum amplitude will not change.

## Determination of the wave breaking field: pseudopotential method

In this section, we derive an analytical expression for the wave breaking limit as a function of input plasma parameters: $\delta _i$, $\beta _{th,e}$, $\mu$, $\gamma _{ph}$ and $\kappa$, using standard pseudopotential approach. Equation (21) can be reduced in the following form

22 $$\begin{aligned} \frac{d^2\phi }{d\psi ^2} + \frac{dU}{d\phi } = 0 \end{aligned}$$

where the potential $U(\phi )$ is given by

23 $$\begin{aligned} U(\phi ) &= V_{ph}\gamma _{ph} ^2 \left[ - (\Phi _1^2 - \gamma _{ph} ^{-2})^{1/2} - \frac{\mu }{Q}(\Phi _2^2 - \gamma _{ph} ^{-2})^{1/2}\right] \\ & \quad + \delta _e V_{ph}\gamma _{ph} ^2 \left[ \frac{\mu }{Q}(\Phi _2^2 - \gamma _{ph} ^{-2})^{1/2} - V_{ph} \phi \right] - \delta _e\beta _{th,e} \\ & \quad \times   \left[ 1 - \frac{\phi }{\beta _{th,e}(\kappa - 3/2)}\right] ^{(-\kappa + 3/2)} + C_1 \end{aligned}$$

Here, $C_1$ is the integrating constant which can be evaluated by considering $U(\phi ) = 0$ at $\phi = 0$ and can be written as

$$\begin{aligned} C_1 = V_{ph} \gamma _{ph}^2 \left( V_{ph} + \frac{\mu }{Q}V_{ph} \right) + \delta _e\beta _{th,e} - \delta _eV_{ph}^2\gamma _{ph}^2\frac{\mu }{Q} \end{aligned}$$

Now, substituting the expression for $C_1$, in Eq. (23) the final form of the pseudopotential stands as

24 $$\begin{aligned}U(\phi ) &= V_{ph}\gamma _{ph}^2\left[ V_{ph} - (\Phi _1^2 - \gamma _{ph} ^{-2})^{1/2} \right] + V_{ph}\gamma _{ph} ^2\frac{\mu }{Q}\left[ V_{ph} - (\Phi _2^2 - \gamma _{ph} ^{-2})^{1/2} \right] \nonumber \\& \quad - \delta _eV_{ph}\gamma _{ph}^2 \left[ \frac{\mu }{Q}V_{ph} - \frac{\mu }{Q}(\Phi _2^2 - \gamma _{ph} ^{-2})^{1/2} +V_{ph}\phi \right] \nonumber \\ & \quad + \delta _e\beta _{th,e} \left[ 1 - \left\{ 1 + \frac{\phi }{\beta _{th,e}(-\kappa + 3/2)} \right\} ^{(-\kappa + 3/2)} \right] \end{aligned}$$

Physically Eq. (22) describes the one dimensional motion of a particle of unit mass in a field with potential $U(\phi )$; the values $\phi$ and $\frac{d\phi }{d\psi }$, respectively, correspond to the position and velocity of this particle. Hence, the whole problem of wave dynamics now can be explained in the framework of the classical mechanical problem of a single particle moving in a nonlinear field, under the potential $U(\phi )$, given by Eq. (24). This pseudopotential provides some insight of the dynamics of the problem. Therefore, a few important characteristics of the wave can be provided by studying the structure of the pseudopotential.

First, we must examine the regime of existence of these waves. It is natural that in order to have a real periodic like solutions, the pseudopotential and the associated fluid variables like density, velocity etc. should be real. This reality conditions impose some constraints on the potential of the wave, since, the terms within the square root of Eq. (24) should be positive. These two constraints are $\Phi _1 \ge \frac{1}{\gamma _{ph}}$ and $\Phi _2 \ge \frac{1}{\gamma _{ph}}$, which ultimately leads to $-\frac{\mu }{Q}\left( 1 - \frac{1}{\gamma _{ph}}\right) \le \phi \le \left( 1 - \frac{1}{\gamma _{ph}}\right)$. These limits are obtained by substituting $\Phi _1 = 1 - \phi$ and $\Phi _2 = 1 + \frac{Q}{\mu }\phi$ in $\Phi _1 \ge \frac{1}{\gamma _{ph}}$ and $\Phi _2 \ge \frac{1}{\gamma _{ph}}$ respectively. Hereafter we denote $-\frac{\mu }{Q}\left( 1 - \frac{1}{\gamma _{ph}}\right)$ and $\left( 1 - \frac{1}{\gamma _{ph}}\right)$ respectively by $\phi _{cr,n}$ and $\phi _{cr,p}$. $\phi _{cr,n}$ and $\phi _{cr,p}$ respectively represents the critical potential values for positive ion and negative ion components, present in the electronegative plasma. Once the potential reaches the critical value, the respective ion density becomes singular at certain point in space. Thus it is not physically possible to generate electrostatic waves beyond this potential range in an electronegative plasma. We note that the constraints on $\phi$ in the classical limit can not be obtained by simply substituting $\gamma _j \rightarrow 1$. The constraints on $\phi$ in the classical limit can be obtained in the following way: In the relativistic limit

25 $$\begin{aligned} \Phi _1 > \frac{1}{\gamma _{ph}} \end{aligned}$$

In the classical limit (when, $v_{ph}^2<< c^2$), $\gamma _{ph} \approx 1 + \frac{v_{ph}^2}{2c^2}$. From Eq. (25)

26 $$\begin{aligned} 1 - \frac{z_1e\phi }{m_1c^2} > 1 + \frac{v_{ph}^2}{2c^2} \end{aligned}$$

Finally Eq. (26) gives

27 $$\begin{aligned} \frac{z_1e\phi }{m_1v_{ph}^2} < \frac{1}{2} \end{aligned}$$

Another constraint on $\phi$ in the classical limit can be found from $\Phi _2 > \frac{1}{\gamma _{ph}}$ in the similar fashion which can be expressed as

28 $$\begin{aligned} \frac{z_1e\phi }{m_1v_{ph}^2} > \frac{\mu }{2Q} \end{aligned}$$

**Figure 5:**
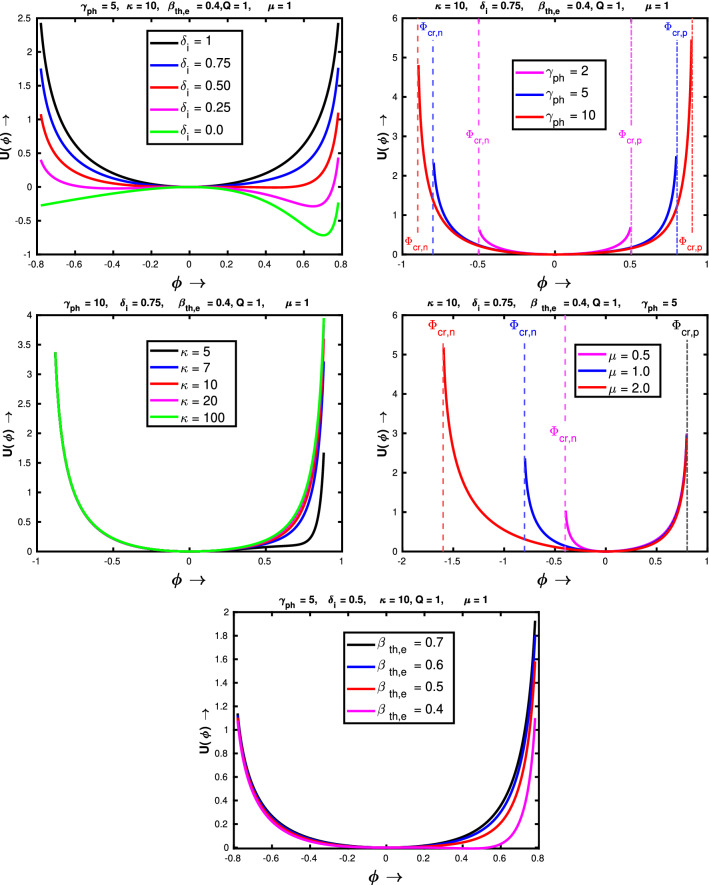
The variation of pseudopotential $U(\phi )$ as a function of $\phi$ for different values of the input parameters: negative-to-positive ion density ratio $\delta _i$ (top left), Lorentz factor $\gamma _{ph}$ (top right), spectral index $\kappa$ (middle left), negative-to-positive ion mass ratio $\mu$ (middle right) and normalised electron thermal energy $\beta _{th,e}$ (bottom).

Now, we investigate the effects of $\delta _i$, $\beta _{th,e}$, $\gamma _{ph}$, $\mu$ and $\kappa$ on the nonlinear pseudopotential. Figure 5 shows the variations of pseudopotential $U(\phi )$ as a function of $\phi$ for an entire range of input parameters. The figure at the top-left of Fig. 5 depicts the influence of negative to the positive ion concentration ratio $\delta _i$ on the pseudopotential. It shows that the pseudopotential becomes more asymmetric and gets wider as the negative ion concentration decreases. Physically it happens due to increase in superthermal electrons. As we can see that for $\delta _i = 1$, there is no superthermal electrons in the system. For $\delta _i = 1$ plasma is composed of only positive and negative ions of equal charges (equal in magnitude but opposite in nature) and masses. As a result the generated pseudopotential is symmetric in the positive and negative side of $\phi$. The reason for widening is associated with the fact that as the concentration of the heavy ions decreases the pseudopotential is more governed by the superthermal electrons. Closer inspection indicates that the widening of the pseudopotential in the positive side of $\phi$ is more as compared to the negative side of $\phi$. It happens due to the dependence of electron density on the potential (Eq. 14). Next, the effect of Lorentz factor $\gamma _{ph}$ has been shown in the top right panel of Fig. 5. It also shows that the potential width increases with the Lorentz factor. It means that the amplitude of the wave increases with the Lorentz factor. For the sake of the reader, we have also pointed out $\phi _{cr, n}$ and $\phi _{cr,p}$ for different values of $\gamma _{ph}$. The pseudopotentials are symmetric on both sides of $\phi$ because for all the three cases $\mu = 1$, therefore $|\phi _{cr, n}| = |\phi _{cr,p}|$. Now, we see the effect of the spectral index $\kappa$ on the pseudopotential which has been shown in middle left panel of Fig. 5. We observe that the width of the pseudopotential curve on the positive side of $\phi$ increases as the spectral index $\kappa$ decreases. But it doesn’t affect the potential on the opposite side i.e. on the negative side of the potential $\phi$^39^. Here also this asymmetry is caused by the power-law dependence of electron density with $\kappa$. We also find that for much higher values of $\kappa$, it hardly effects the structure of the pseudopotential since for higher $\kappa$ value the distribution becomes Maxwellian. The effect of negative to positive ion mass $\mu$ on the pseudopotential has been presented in the middle right panel of Fig. 5. In this figure the asymmetry in the pseudopotential actually arises when the value of $\mu$ deviates from unity. For $\mu < 1$, which is shown by magenta colour, the pseudopotential is much wider on the positive side of $\phi$, since $|\phi _{cr,n}| < \phi _{cr,p}$. On the other hand, for $\mu > 1$, which is shown by red colour, the pseudopotential is much wider on the negative side of $\phi$, since $|\phi _{cr,n}| > \phi _{cr,p}$. For $\mu = 1$, the pseudopoptential is stretched equally in both directions, because for $\mu = 1$, $|\phi _{cr,n}| = \phi _{cr,p}$. The values of $\phi _{cr,n}$ for three different values of $\mu$ has been shown in respective colours. Note that $\phi _{cr,p}$ has been marked with black colour only, as it is independent of $\mu$. Now finally we show the impact of electron temperature on the pseudopotential in the bottom panel of Fig. 5. We find that, for fixed values of other variables, the pseudopotential gets wider and asymmetric as the $\beta _{th,e}$ decreases. Here this effect is primarily caused by the term $\frac{\phi }{\beta _{th,e}(\kappa - 3/2)}$. As we can see for fixed values of other parameters this term increases as $\beta _{th,e}$ decreases.

Now. we proceed to determine the maximum electric field amplitude sustained by these waves. We integrate Eq. (22), which simply gives the following form

29 $$\begin{aligned} \frac{1}{2}\left[ \frac{d\phi }{d\psi } \right] ^2 + U(\phi ) = C_2 \end{aligned}$$

where, $C_2$ is an another integration constant, which can be determined by considering the condition that at $\phi = 0$, $\frac{d\phi }{d\psi }$ is extremum ($E_{max}$), since $\frac{d\phi }{d\psi }$ is the normalised electric field. Therefore, from Eq. (29)

30 $$\begin{aligned} \frac{1}{2}\left[ \frac{d\phi }{d\psi } \right] ^2 + U(\phi ) = \frac{E_{max}^2}{2} \end{aligned}$$

Now, when $\phi$ attains its maximum allowed value ($\phi _{max}$), $\frac{d\phi }{d\psi }$ vanishes. Thus, the expression for the wave breaking field finally stands as

31 $$\begin{aligned} E_{WB} = \sqrt{2U_{max}} \end{aligned}$$

beyond this amplitude the wave will break within a period and the wave coherence will be destroyed ultimately. As a result, at the point of wave breaking, the wave energy is converted to random particle energy leading to efficient particle acceleration and plasma heating. Now, $U(\phi )$ will become maximum at critical values of the potentials; either at $\phi _{cr,p}$ or at $\phi _{cr,n}$ (or, at both points), depending upon the value of $\frac{\mu }{Q}$. For $\frac{\mu }{Q} > 1$, $|\phi _{cr,n}| > \phi _{cr,p}$; therefore, periodic solutions are possible up to $U_{max}(\phi _{cr,p})$. As a result, the wave breaking field for electronegative plasma with $\frac{\mu }{Q} > 1$ will be

32 $$\begin{aligned} E_{WB, \frac{\mu }{Q} > 1} = \sqrt{2U_{max}(\phi _{cr,p})} \end{aligned}$$

This wave breaking limit is manifested by the density singularity of the positive ion species, since negative ions have higher mass. On the other hand for negative ion species with $\frac{\mu }{Q} < 1$, $|\phi _{cr,n}| < \phi _{cr,p}$; therefore, periodic bound state solutions are possible up to $U_{max}(\phi _{cr,n})$. As a result, the wave breaking field for electronegative plasma with $\frac{\mu }{Q} < 1$ stands as

33 $$\begin{aligned} E_{WB, \frac{\mu }{Q} < 1} = \sqrt{2U_{max}(\phi _{cr,n})} \end{aligned}$$

**Figure 6:**
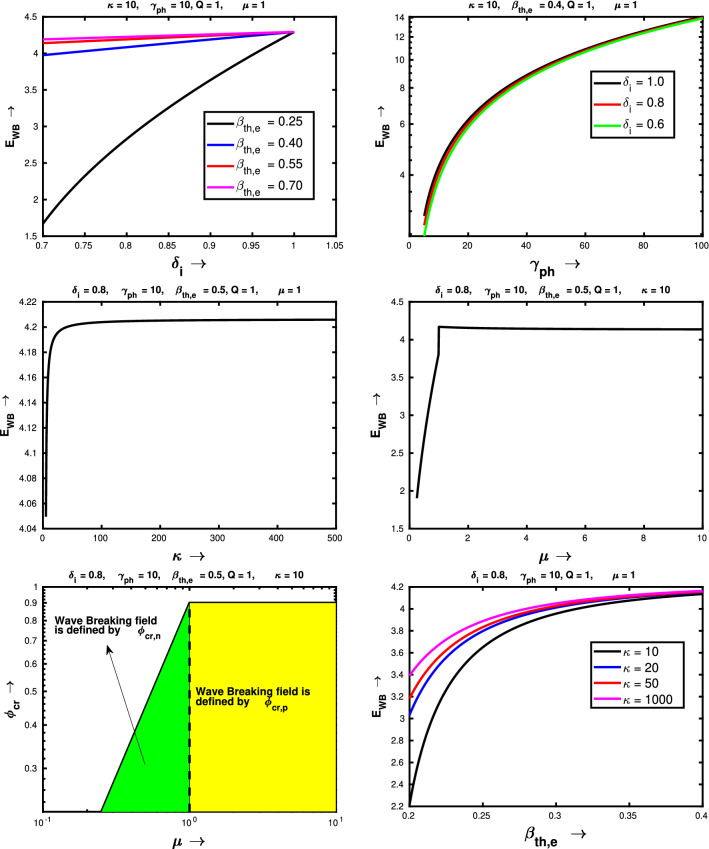
The variation of wave breaking amplitude with the negative-to-positive ion density ratio $\delta _i$ (top left), Lorentz factor $\gamma _{ph}$ (top right), spectral index $\kappa$ (middle left) and negative-to-positive ion mass ratio $\mu$ (middle right). Variation of critical value of $\phi$ as a function of $\mu$ (bottom left) and variation of wave breaking amplitude with the normalised electron thermal energy $\beta _{th,e}$ (bottom right).

Physically speaking, the wave breaking amplitude, presented by Eq. (33) is attributed to the density singularity of the secondary negative ion species, present in the plasma. Therefore, depending upon the electronegative plasma composition only one critical value should be chosen in order to obtain the wavebreaking field. Now we show the variation of wave breaking field of relativistically intense waves in a electronegative plasma, given by Eqs.(32) and (33) with various input plasma parameters, which essentially govern the dynamics of the wave and the pseudopotential. Figure 6 shows the effect of various input parameters on the wave breaking limit, for fixed values of other parameters. From the top left panel we see that, the wave breaking field essentially increases with the concentration of negative ion species which also happens for nonrelativistic electronegative plasma case^39^ and electron-ion-positron plasma case^33^. In Ref.^39^ it has been mentioned that to that the reason behind this enhancement may be due to the increase of the Debye length due to the presence of negative ions. This result has been shown for four different initial values of electron temperature. We see that for $\delta _i = 1$ i.e. for $\delta _e = 0$, wave breaking limit is independent of $\beta _{th, e}$. It is natural to expect because for $\delta _e = 0$, motion is not governed by the electrons anymore.

Next, we show the variation of wave breaking limit with the relativistic Lorentz factor on the top right corner of Fig. 6. In this figure, we find that the wave breaking limit is increasing with $\gamma _{ph}$, which is natural to expect for relativistic waves in laser plasma interaction scenario^22,24,33,34,37,56^. The variation of $E_{WB}$ with spectral index of electron velocity distribution has been presented in the left middle panel of Fig. 6. Here, we observe that, for lower values of $\kappa$, wave breaking limit rises rapidly with $\kappa$ as the superthermal population decreases with increasing $\kappa$ and for $\kappa \approx 100$, it saturates. We believe it happens due to the effect that for higher $\kappa$ values, the kappa distribution will become a Maxwellian distribution and doesn’t change even after increasing the value of $\kappa$. Now, we show the effect of $\mu$ on $E_{WB}$ for a electronegative plasma. From the right middle panel of Fig. 6 we see that for $\mu < 1$, $E_{WB}$ increases with $\mu$ and in the regime $\mu > 1$, it is independent of $\mu$. To explain this physical nature we have shown critical values of $\phi$ as a function of $\mu$, which has been depicted in the left bottom panel of Fig. 6. We note that, in this plot $Q = 1$, therefore for $\mu < 1$, $E_{WB}$ is defined in terms of $|\phi _{cr,n}|$ which increases with $\mu$ (shown by the green patch). On the othet hand, for $\mu > 1$, $E_{WB}$ is given by $|\phi _{cr,p}|$ which is independent of $\mu$ (shown by the yellow patch). Therefore $E_{WB}$ increases with $\mu$ until $\frac{\mu }{Q}$ reaches unity, after that $E_{WB}$ is independent of $\mu$ as in this domain $|\phi _{cr,p}|$ is independent of $\mu$. Finally, we show the variation of wave breaking field with $\beta _{th,e}$ in the right bottom panel of Fig. 6. It shows that initially the wave breaking field rises with increasing $\beta _{th,e}$. But, for $\beta _{th,e} > 0.5$, slowly the wave breaking field reaches to an asymptotic value (close to 4.2).

Here we note that, by treating the electrons as relativistic fluid instead of superthermal components and positive and negative ions respectively as positrons and secondary ions, we can reproduce the results obtained by Karmakar et al.^33^. We, note that in order to derive the results of Karmakar et al., the quasinuetrality condition [given by Eq. (8)] should be applied properly, since in that case^33^ the equilibrium electron density is equal to the sum of equilibrium proton and ion densities. But in our present study, equilibrium positive ion density is equal to the sum of equilibrium negative ion density and equilibrium superthermal electron density. On the other hand, we can also reach to the Akhiezer-Polovin limit ($E_{AP}$)^24^ by treating the electrons as relativistic cold fluid while the ions are fixed neutralizing background, proving the nonlinear restoring force.

Finally, we calculate the wavelength of these waves and study its dependence on the different input parameters. The wavelength for this nonlinear waves can be estimated as the twice the distance between the extremum potential points^33,57^. Therefore in normalised unit $\left[ \left( \frac{c}{\omega _{p1}}\right) ^{-1}\right]$, the expression for wavelength can be expressed as

34 $$\begin{aligned} \lambda _P = 2\times \int _{\phi _{min}} ^{\phi _{max}} \frac{d\phi }{\left( \frac{d\phi }{d\psi }\right) } \end{aligned}$$

In the above equation $\phi _{min}$ and $\phi _{max}$ are two roots of where $\frac{d\phi }{d\psi } = 0$^33,57^. We have calculated these roots from Eq. (30) after solving for $\frac{d\phi }{d\psi } = 0$. We have carefully noted these $\phi$ values where $\frac{d\phi }{d\psi } = 0$ and then numerically integrated Eq. (34) to calculate the value of the wavelength. We have repeated this experiment for different sets of input parameters in order to get the dependence of wavelength on the input plasma parameters, which govern the dynamics of the wave.

**Figure 7:**
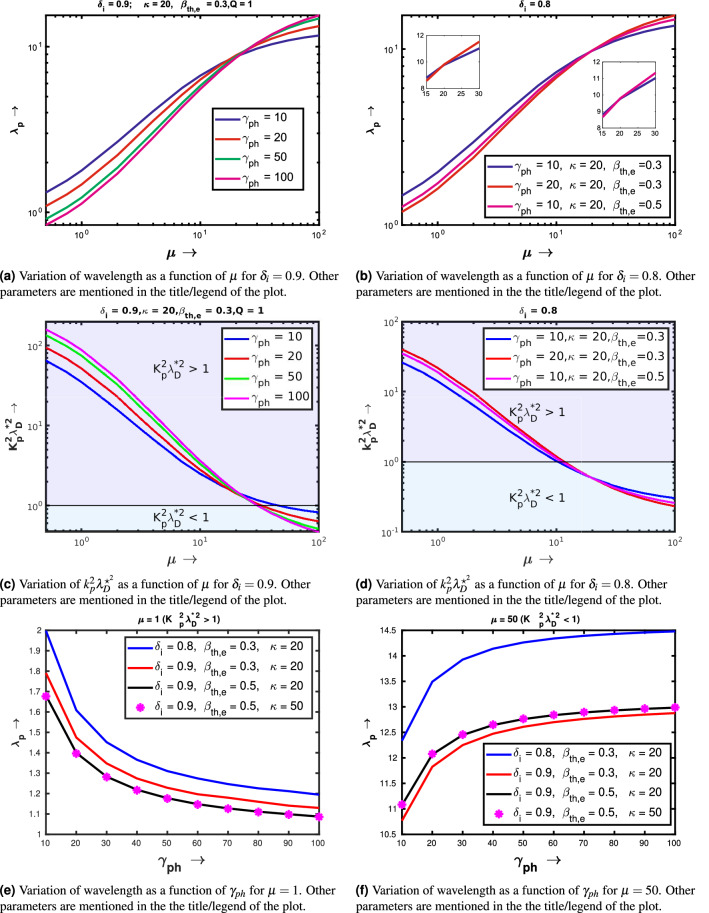
Variation of wavelength ($\lambda _P$) and ossociated $k_p^2\lambda _D^{\star ^2}$ for relativistic electrostatic waves in an electronegative plasma with the negative to positive ion mass ratio and Lorentz factor.

Plots in Fig. 7 show the variation of nonlinear wavelength on $\mu$ and $\gamma _{ph}$. Figure 7a first shows the dependence of $\lambda _p$ on $\mu$ for different values of $\gamma _{ph}$. The following input parameters have been kept constant: $\delta _i = 0.9, \kappa = 20, \beta _{th,e} = 0.3$ and $Q=1$. We find that the wavelength increases with $\mu$. This increment is associated with out-of-phase motion between the positive ion and negative ion fluid (see Figs. 3a,b and 4a; the maxima of the positive ion fluid density occurs at the minima of the negative ion fluid density and vice versa). In this figure we also observe that for $\mu \le 20$, the wavelength of the wave decreases with $\gamma _{ph}$. There also exists a transition region at $\mu \approx 20$, after that the wavelength increases with the Lorentz factor. The same trend have also been obtained by changing the value of $\delta _i =0.8$, which has been shown in Fig. 7b. Here also the wavelength increases with $\mu$. By comparing the blue and red curves in Fig. 7b, we observe that $\mu \le 20$, the wavelength of the wave decreases with $\gamma _{ph}$ and after that the wavelength increases with $\gamma _{ph}$. For the sake of the readers, the transition region $\mu \approx 20$ has been shown in the left inset of Fig. 7b. Another transition has been seen by changing the value of $\beta _{th,e}$. From the blue and magenta graph we find that for the values of $\mu \le 20$, the wavelength essentially decreases with $\beta _{th,e}$ and for $\mu > 20$, the wavelength increases with $\beta _{th,e}$. For the aiding of the eye, this transition with $\beta _{th,e}$ has been been shown in the right inset plot of Fig. 7b.

In order to understand this dramatic behaviour, in Fig. 7c,d, we have shown the variation of $k_p^2\lambda _D^{\star ^2}$ as a function of $\mu$ for the other input parameters as taken in Fig. 7e,f respectively. From, Fig. 7c we see that, as the ratio of negative to positive ion mass increases, the value of $k_p^2\lambda _D^{\star ^2}$ decreases significantly. In fact, for higher $\mu$ values ($> 30$) there is a transition from $k_p^2\lambda _D^{\star ^2} > 1$ regime to $k_p^2\lambda _D^{\star ^2} < 1$ regime, which have been marked by two different background colors. $k_p^2\lambda _D^{\star ^2} = 1$ has been shown by horizontal black line. From this graph, we understand that the variation of wavelength with $\gamma _{phi}$ is different in both two regimes. For $k_p^2\lambda _D^{\star ^2}>> 1$ (when the wavelengths are less than the kappa dependent Debye screening length), the wavelength decreases with the Lorentz factor; on the other hand for $k_p^2\lambda _D^{\star ^2}<< 1$ (when the wavelengths are higher than the kappa dependent Debye screening length), the wavelength increases with the Lorentz factor. In the similar way, Fig. 7d also explains the similar behaviour with $\beta _{th,e}$ in both two regions. Again from Fig. 7d, we see that the transition occurs near $k_p^2\lambda _D^{\star ^2} = 1$. In the regime, where $k_p^2\lambda _D^{\star ^2}>> 1$ (for nonlinear ion plasma oscillations), the nonlinear wavelength decreases with $\beta _{th,e}$ and the parameter domain where $k_p^2\lambda _D^{\star ^2}<< 1$ (for nonlinear ion acoustic modes), the wavelength essentially increases with $\beta _{th,e}$.

To confirm our previous analysis finally we show the variation of wavelength as a function of $\gamma _{ph}$, for $\mu = 1$ ($k_p^2\lambda _D^{\star ^2}>> 1$) and $\mu = 50$ ($k_p^2\lambda _D^{\star ^2}<< 1$). Figure 7e,f respectively show the wavelength as a function of Lorentz factor for $\mu = 1$ and $\mu = 50$. In each figures the colour codes have been mentioned explicitly inside the legend. From these two figures, we find that for smaller values of $\mu$ when $k_p^2\lambda _D^{\star ^2}>> 1$, the wavelength decreases with $\gamma _{ph}$ and for relatively higher values of $\mu$, when $k_p^2\lambda _D^{\star ^2}<< 1$, wavelength increases with $\gamma _{ph}$. In both two figures, by comparing the blue and red curves, we can also conclude that, an increment in negative ion fraction, essentially leads to a reduction in the wavelength of the wave. Another interesting point has been re-confirmed by comparing the red ($\beta _{th,e} = 0.3$) and black graphs ($\beta _{th,e} = 0.5$) that for $\mu = 1$ when $k_p^2\lambda _D^{\star ^2}>> 1$, the wavelength decreases with $\beta _{th,e}$, whereas for for $\mu = 50$ when $k_p^2\lambda _D^{\star ^2}<< 1$, the wavelength increases with $\beta _{th,e}$. The same experiment has been repeated by changing the value of $\kappa$ from 20 to 50, for $\mu = 1$ and $\mu = 50$, which have been depicted via magenta dots. By comparing the magenta graph with black one we may observe that the wavelengths in the nonlinear regime are almost independent of the spectral index $\kappa$.

## Conclusions

In conclusion, the nonlinear dynamics of relativistically intense electrostatic waves in an unmagnetised electronegative plasma has been studied, where the electron velocity distribution can be modelled as a $\kappa$ distribution function. A relativistic nonlinear multi-fluid model has been adopted to depict the self sustained dynamics of these waves in a stationary wave frame. The equation of motion has been derived and its numerical solutions have been presented. The parameter domain where these waves are likely to be observed has been delineated explicitly. Studies have been further extended to discover the maximum electric field amplitude sustained by these nonlinear waves, using standard Sagdeev pseudopotential approach. It has also been found that the wave breaking field actually increases with the fraction of negative ion species and also with the Lorentz factor. The influence of spectral index of electron velocity distribution, negative to positive ion mass ratio and electron thermal energy on the wave breaking limit have also been shown, for fixed values of other input parameters. From the structure of the pseudopotential, the wave breaking point has been figured out, which may manifested by the density singularity either of primary positive ion fluid (for $\frac{\mu }{Q} > 1$) or of secondary negative ion fluid (for $\frac{\mu }{Q} < 1$) , depending upon the plasma composition. Thus in terms of trajectory crossing we may conclude that, wave breaking occurs due to the trajectory crossings either of the primary positive ion species when $\frac{\mu }{Q} > 1$ or of the secondary negative ion species when $\frac{\mu }{Q} < 1$. This trajectory crossing destroys the coherent motion of the oscillating particles, constituting the wave. As a result the energy which was loaded to excite coherent oscillation of the plasma particles gets converted to their random motion. In kinetic picture at the onset of wave breaking point, for $\frac{\mu }{Q} > 1$ the positive ion species will be trapped whereas the negative ions will be trapped when $\frac{\mu }{Q} < 1$. The fraction of trapped ions increases with the potential and hence with the electric field amplitude^34,35^. In our study we found that the wave breaking limit increases as the fraction of secondary ion increases. Naturally a larger field can trap a higher fraction of particles (lighter ions). Thus from our theory a larger amount of trapping and hence acceleration are expected in the presence of secondary ion species. Similar results have also been obtained from Particle-in-Cell simulations published earlier. For example, In Ref.^37^ it has been found that proton trapping and acceleration get enhanced when there is a sufficient amount of heavier ions like Tritium present in plasma. Another relevance can be found in Ref.^35^, where the trapping of protons and their acceleration increase due the presence of heavy ions mixture.

The wavelengths of these nonlinear waves have also been calculated and its dependence on the various input parameters like fraction of negative ion species, negative to positive ion mass ratio, Lorentz factor and electron temperature has been presented. It has been found that the wavelength decreases with the negative ion concentration and increases with negative to positive ion mass ratio. Moreover, it has also been shown that for $\mu < 20$ (when $k_p^2\lambda _D^{\star ^2}>> 1$, corresponding to the nonlinear ion plasma oscillations), the wavelength decreases with the Lorentz factor and electron temperature. Then there exist a transition region near the point $k_p^2\lambda _D^{\star ^2} = 1$ and after that transition when $k_p^2\lambda _D^{\star ^2}<< 1$ (corresponding to the nonlinear ion acoustic mode) the wavelength increases the Lorentz factor and electron temperature. These results may be relevant in understanding the dynamics of relativistic waves in space and laboratory experiments where multi-ion species plasma or electronegative plasma can exist.

In addition, our results provide a prior estimation on the wave breaking limit of relativistic electrostatic waves in an electronegative plasma. The numerical solutions given in this manuscript can be used as an initial condition to excite waves in PIC simulations to study the stability of the waves and acceleration mechanism via wave breaking in a electronegative plasma. Our model might be helpful in self injection schemes^34^ in a multi component plasma where a prior knowledge of wave breaking field is necessary during the excitation of the wave.

## Data Availability

All data generated or analysed during this study are included in this published article.
